# Extrapulmonary Nontuberculous Mycobacterial Disease Surveillance — Oregon, 2014–2016

**DOI:** 10.15585/mmwr.mm6731a3

**Published:** 2018-08-10

**Authors:** David C. Shih, P. Maureen Cassidy, Kiran M. Perkins, Matthew B. Crist, Paul R. Cieslak, Richard L. Leman

**Affiliations:** ^1^Epidemic Intelligence Service, CDC; ^2^Public Health Division, Oregon Health Authority, Portland, Oregon; ^3^National Center for Emerging and Zoonotic Infectious Diseases, Division of Healthcare Quality Promotion, CDC.

## Abstract

Nontuberculous mycobacteria (NTM), ubiquitous in soil and water, usually infect immunocompromised persons. However, even healthy persons are susceptible to infection through percutaneous inoculation. Although 77% of NTM diseases manifest as primarily pulmonary illnesses (*1*), NTM also infect skin, bones, joints, the lymphatic system, and soft tissue. NTM infections can have incubation periods that exceed 5 years (*2*), often require prolonged treatment, and can lead to sepsis and death. Extrapulmonary NTM outbreaks have been reported in association with contaminated surgical gentian violet (*3*), nail salon pedicures (*4*), and tattoos received at tattoo parlors (*5*), although few surveillance data have been available for estimating the public health burden of NTM.* On January 1, 2014, the Oregon Health Authority designated extrapulmonary NTM disease a reportable condition. To characterize extrapulmonary NTM infection, estimate resources required for surveillance, and assess the usefulness of surveillance in outbreak detection and investigation, 2014–2016 extrapulmonary NTM surveillance data were reviewed, and interviews with stakeholders were conducted. During 2014–2016, 134 extrapulmonary NTM cases (11 per 1 million persons per year) were reported in Oregon. The age distribution was bimodal, with highest incidence among persons aged <10 years (20 per 1 million persons per year) and persons aged 60–69 years (18 per 1 million persons per year). The most frequently reported predisposing factors (occurring within 14–70 days of symptom onset) were soil exposure (41/98; 42%), immunocompromised condition (42/124; 34%), and surgery (32/120; 27%). Overall, 43 (33%) patients were hospitalized, 18 (15%) developed sepsis, and one (0.7%) died. Surveillance detected or helped to control two outbreaks at low cost. Jurisdictions interested in implementing extrapulmonary NTM surveillance can use the Council of State and Territorial Epidemiologists (CSTE) standardized case definition (*6*) for extrapulmonary NTM reporting or investigative guidelines maintained by the Oregon Health Authority (*7*).

In Oregon, electronic laboratory reports of reportable diseases are uploaded daily to the statewide communicable disease database, the Oregon Public Health Epidemiologists’ User System (Orpheus). Staff members from the patients’ local public health jurisdiction investigate extrapulmonary NTM cases by collecting clinical data and information on any predisposing factors occurring during the 14–70 days preceding symptom onset from medical charts and patient interviews, then enter the data into Orpheus. An epidemiologist reviews case data for quality and completeness and generates annual state infectious disease epidemiology reports. The Oregon Health Authority does not require laboratories to retain extrapulmonary NTM isolates.

For this analysis, a case of extrapulmonary NTM was defined (according to Oregon Health Authority investigative guidelines at the time) as a culture-confirmed extrapulmonary NTM infection involving skin or soft tissue from a wound or abscess, lymphatic tissue, urine, or other normally sterile site (e.g., blood or spinal fluid), in an Oregon resident, with the first specimen collected during January 1, 2014–December 31, 2016, and extrapulmonary NTM symptom onset after December 31, 2012. Cultures that were positive only for *Mycobacterium gordonae*, a common environmental contaminant, were excluded. Patient demographics and predisposing factors (prespecified by literature review and expert opinion) were described, and incidence was calculated using 2014–2016 Oregon population estimates from the Portland State University Population Research Center. Resource requirement estimates were developed through interviews with stakeholders, including the Oregon Health Authority epidemiologist whose assignments include extrapulmonary NTM surveillance, the informatics programmer, and three local public health nurses who estimated public health personnel time to perform extrapulmonary NTM surveillance. The utility assessment consisted of a review of how extrapulmonary NTM surveillance data were used to identify or investigate outbreaks.

## Characteristics of Extrapulmonary NTM Cases

During 2014–2016, a total of 134 extrapulmonary NTM cases were reported in Oregon (11 per 1 million persons per year). Patients ranged in age from 10 months to 92 years (median age = 50.8 years). Seventy (52%) patients were female, 96 (72%) were white, 43 (33%) were hospitalized, 18 (15%) developed sepsis, and one (1%) died. Among patients for whom exposure risk factors were reported, the most frequently reported predisposing factors were soil exposure (41/98; 42%), immunocompromised condition (42/124; 34%), and surgery (32/120; 27%). Approximately two thirds of patients (68%) reported more than one predisposing factor (Table 1). A bimodal age distribution was observed, with highest number of cases and the highest incidence among persons aged 0–9 years (20 per 1 million persons per year) and persons aged 60–69 years (18 per 1 million persons per year) (Table 2). Among 29 infections in patients aged 0–9 years, 25 (86%) were caused by *Mycobacterium avium* complex. Among 26 infections in patients aged 60–69 years, six (23%) were caused by *Mycobacterium avium* complex. The remainder of cases in this age group primarily was caused by either *M. chelonae* or *M. abscessus* (nine; 35%) or *M. fortuitum* (six; 23%) (Figure).

**TABLE 1:** Characteristics, clinical outcomes, and predisposing factors for 134 cases of extrapulmonary nontuberculous mycobacteria (NTM) infections — Oregon, 2014–2016

Characteristic	No. (%)
**Total cases 2014–2016**	**134 (100)**
2014	45 (34)
2015	44 (33)
2016	45 (34)
**Sex**	
Female	70 (52)
Male	64 (48)
**Race**	
White	96 (72)
Asian/Pacific Islander	9 (7)
Other or multiple	6 (4)
Black	2 (1)
American Indian/Alaska Native	1 (1)
Unknown	20 (15)
**Ethnicity**	
Non-Hispanic	96 (72)
Hispanic	12 (9)
Unknown	26 (19)
**Outcome**	
Hospitalized (130)	43 (33)
Sepsis (123)	18 (15)
NTM-related death	1 (1)
Predisposing factor*	
Worked with soil (98)	41 (42)
Immunocompromised (124)	42 (34)
Surgery (120)	32 (27)
Outpatient infusions or injections (110)	24 (22)
Skin trauma (107)	21 (20)
Immunosuppressive therapy (120)	23 (19)
Hot tub or spa use (104)	16 (15)
Acupuncture (106)	13 (12)
Fish tank maintenance (104)	9 (9)
Nail salon visit (103)	7 (7)
Fish handling (105)	6 (6)
Tattoo receipt (108)	2 (2)
>1 Predisposing factor^†^ (124)	84 (68)

* 14–70 days before symptom onset.

^†^ Denominator for >1 predisposing factor row is the total number of patients who responded to at least two questions.

**TABLE 2:** Number of extrapulmonary nontuberculous mycobacteria (NTM) cases and incidence, by year and age group — Oregon, 2014–2016

Year/Age group	No. of cases	Cases per 1 million persons per year
**Overall**	**134**	**11**
**Year**		
2014	45	11
2015	44	11
2016	45	11
**Age group (yrs)**		
0–9	29	20
10–19	2	1
20–29	3	2
30–39	10	6
40–49	21	13
50–59	26	16
60–69	26	18
70–79	13	16
80–99	4	8

**FIGURE:**
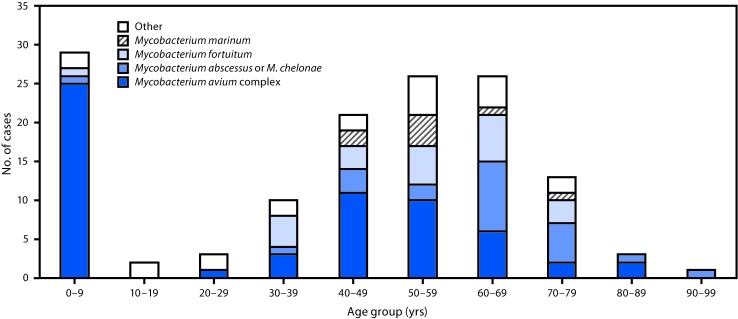
Nontuberculous mycobacteria (NTM) species identified in cases with extrapulmonary NTM infections, by age group — Oregon, 2014–2016

Among persons aged 0–9 years, 76% had infected lymph nodes, compared with 4% among persons aged 60–69 years. The latter age group’s most common specimen sources were tissue (31%) (not further specified), wounds (19%), blood (12%), and joints (12%).

## Detection and Management of Extrapulmonary NTM Outbreaks

An outbreak of seven *M. fortuitum* infections in two small neighboring counties was initially reported by hospital staff members after the outbreak had begun in July 2013 and before mandatory reporting had commenced in January 2014. The outbreak was associated with knee and hip replacement procedures during 2013–2014, a single device manufacturer, and multiple hospitals and operating room staff members. The investigation began 102 days after the second confirmed laboratory report became available. Intraoperative risks identified included suboptimal surgical infection control practices. The presence of a single device manufacturer representative^†^ at six of the seven surgical procedures was associated with NTM surgical site infection. Oregon Health Authority received no reports of cases of *M. fortuitum* infections among joint replacement patients beyond the two counties. Public health officials recommended that all operating room staff members adhere to the Association of Perioperative Registered Nurses infection control guidelines.

During 2015, two *M. haemophilum* infections were reported to a local health department; both involved receipt of tattoos at the same tattoo parlor. The outbreak investigation began 33 days after the second confirmed laboratory report became available. The tattoo artist used water from a cooler to dilute ink and wipe tattoos during their placement. After public health officials recommended using sterile water, no additional extrapulmonary NTM infections were associated with the parlor.

## Costs of Establishment and Maintenance of Extrapulmonary NTM Surveillance

All estimated costs related to extrapulmonary NTM surveillance were for salaries. Local health department nurses reported spending approximately 90 minutes investigating each case. Incremental direct costs to add extrapulmonary NTM to public health notifiable disease surveillance were approximately $6,000 for implementation and approximately $10,000 in annual operating costs. 

## Discussion

During 2005–2006, an analysis of Oregon laboratory data reported an annual extrapulmonary NTM infection prevalence of 16 cases per 1 million persons (*1*); however, clinical data were insufficient to characterize disease burden, and the authors reported only prevalence, not incidence data. In January 2014, extrapulmonary NTM infections became reportable in Oregon. Although extrapulmonary NTM infections are rare, they can be associated with substantial severity, including hospitalization, sepsis, and death. Costs to set up and maintain the surveillance system were minimal. Limited time was needed to investigate each case, case counts were few, and existing electronic reporting infrastructure minimized laboratory reporting costs.

In Oregon, extrapulmonary NTM surveillance detected outbreaks, augmented case finding, and guided subsequent control measures. Surveillance aided the outbreak investigation among joint replacement patients; the lack of cases reported elsewhere in the state argued against widespread product contamination during manufacturing. That is, because NTM was reportable in Oregon, surveillance would have identified extrapulmonary NTM infections among joint replacement patients in other counties if a production site contaminated the products. Surveillance for extrapulmonary NTM infections also detected the outbreak among tattoo parlor patrons who lacked a common health care provider who might have recognized a pattern and reported the outbreak. Time to investigation of the tattoo parlor–associated outbreak was 69 days shorter than the time to investigate the previous outbreak that began before mandatory extrapulmonary NTM reporting. If the outbreak among joint replacement patients had occurred when reporting and surveillance procedures were established, the investigation might have begun sooner.

Detection of extrapulmonary NTM outbreaks can be delayed if the condition is not reportable. For example, NTM is not reportable in Georgia. Investigation of an outbreak of extrapulmonary *M. abscessus* infections after dental pulpotomy in Georgia commenced approximately 1 year after the second case was diagnosed; 20 cases among children were ultimately identified (*8*) (Melissa Tobin-D’Angelo, Georgia Department of Public Health, personal communication, June 2018). 

It is important for clinicians to be aware of the possibility of an NTM outbreak because they can help identify extrapulmonary NTM outbreaks. In 2013, a clinician reporting two extrapulmonary NTM cases among medical tourists led to detection of an NTM outbreak traced to cosmetic surgery centers in the Dominican Republic; subsequent case finding identified outbreak cases from four other states (*9*,*10*). Extrapulmonary NTM surveillance could enhance detection and identification of the source of multijurisdictional outbreaks. Contaminated cardiopulmonary bypass heater-cooler devices have caused a large, ongoing international outbreak of *M. chimaera* infections among cardiac surgery patients (*2*). Long incubation periods complicated detection of this outbreak. Systematic extrapulmonary NTM surveillance in other states and countries might have led to earlier detection.

The findings in this report are subject to at least three limitations. First, the routinely asked predisposing factor questions did not specify whether a particular factor (e.g., surgery) involved the infection site, which could have resulted in overestimates of that factor’s impact. In January 2018, the case report form was revised to address this issue. Second, sensitivity of extrapulmonary NTM surveillance might be limited because clinicians might not suspect extrapulmonary NTM infection and, consequently, might not order cultures for mycobacteria. Finally, these data only represent cases diagnosed in Oregon during 2014–2016 and are not generalizable to other states because of different population characteristics, predisposing factor rates, and adoption of electronic laboratory reporting.

To promote nationwide extrapulmonary NTM surveillance, CSTE developed a standardized case definition for extrapulmonary NTM surveillance (*6*). State and territorial public health authorities can use this case definition to ensure compatible surveillance across jurisdictions. In addition, the Oregon Health Authority improved its investigative guidelines and case report form by making the predisposing factor questions body-site specific. Forms are publicly available for states and territories to adapt for extrapulmonary NTM surveillance implementation (*7*). NTM surveillance is ongoing in Oregon. 

Extrapulmonary NTM infections cause considerable morbidity, sometimes resulting in hospitalization or sepsis, in Oregon. Systematic reporting of these infections has led to detection and control of outbreaks at relatively low cost. Publicly available resources (e.g., the CSTE case definition, Oregon’s investigative guidelines, and the Oregon case report form) offer states and territories adaptable tools to implement extrapulmonary NTM surveillance.

**Summary**

**What is already known about this topic?**

Nontuberculous mycobacteria (NTM) infections can cause serious morbidity, especially in health care–associated infections and outbreaks.

**What is added by this report?**

Oregon instituted mandatory extrapulmonary NTM reporting in January 2014. During 2014–2016, 134 cases were reported (11 cases per 1 million persons per year), including 43 hospitalizations, 18 patients with sepsis, and one death. The surveillance system helped detect or control two outbreaks at low cost.

**What are the implications for public health practice?**

Publicly available resources (e.g., the Council of State and Territorial Epidemiologists case definition, Oregon’s investigative guidelines, and the Oregon case report form) offer states and territories adaptable tools to implement surveillance for extrapulmonary NTM infections.

## Footnotes

† Because of prosthetic joints’ complexity, device manufacturer representatives commonly attend and advise the surgeon during joint replacement surgeries.
